# Evaluation of Stability of Surface-Treated Mini-Implants in Diabetic Rabbits

**DOI:** 10.1155/2014/838356

**Published:** 2014-05-28

**Authors:** Nam-Hee Oh, Eun-Young Kim, Janghyun Paek, Yoon-Ah Kook, Do-Min Jeong, Il-Sik Cho, Gerald Nelson

**Affiliations:** ^1^Department of Orthodontics, The Catholic University of Korea, Seoul, Republic of Korea; ^2^Department of Pediatrics, School of Medicine, Chosun University, 30-1 Hak-dong, Dong-gu, Gwangju 501-717, Republic of Korea; ^3^Department of Prosthodontics, School of Dentistry, Kyung Hee University, Hoegi-dong, Dongdaemun-gu, Seoul 130-701, Republic of Korea; ^4^Department of Dentistry, National Medical Center of Korea, 245 Euljiro, Jung-gu, Seoul 100-799, Republic of Korea; ^5^Department of Dentistry, Korea University, 80 Guro-dong, Guro-gu, Seoul 152-703, Republic of Korea; ^6^Division of Orthodontics, School of Dentistry, University of California San Francisco, 707 Parnassus Avenue, San Francisco, CA 94143, USA

## Abstract

*Introduction*. The purpose of this study was to investigate effects of surface treatment of mini-implants in diabetes-induced rabbits by comparing osseointegration around mini-implants. *Methods*. Twelve New Zealand white rabbits were divided into two groups (alloxan-induced diabetic group and control group). A total of 48 mini-implants were placed after four weeks of diabetic induction. 24 mini-implants were surface-treated with SLA (sandblasted with large grit, and acid etched) and the remaining 24 mini-implants had smooth surfaces. Four weeks after placement, 32 mini-implants were removed from 4 control and 4 diabetic rabbits. Insertion and removal torques were measured. The remaining 16 mini-implants from the two groups were histomorphometrically analyzed. *Results*. Maximum insertion torque showed no difference between diabetic and control groups, but total insertion energy was higher in control group. In surface-treated mini-implants, maximum removal torque was higher in both diabetic and control groups. Bone-implant contact (BIC) was increased in the control group when compared to the diabetic group. Surface-treated group had higher BIC than smooth surface group in both control and diabetic groups. However, there was no significantly statistical difference. *Conclusions*. Type 1 diabetes mellitus and surface treatment method of mini-implant affected primary stability of mini-implants. In addition, the use of orthodontic mini-implants in a diabetic patient is likely to show results similar to the healthy patient.

## 1. Introduction

Use of orthodontic mini-implants is gaining popularity due to providing absolute anchorage without reactive tooth movement. With skeletal anchorage such as osseous dental implants, miniplates, miniscrews, or microscrews, clinicians can expect reliable anchorage while depending less on patient compliance [1–7]. Most current orthodontic mini-implants have untreated screw surfaces, and mini-implants achieve primary stability through mechanical retention [5, 8]. In the field of prosthodontics, surface-treated dental implant is preferred due to its improved osseointegration [9–12]. A few surface-treated orthodontic mini-implants are available and their removal torque is higher than smooth surface mini-implant. Surface-treated mini-implants are reported to have less failure and can support heavier and more dynamic forces [13, 14].

Recently, the number of adult orthodontic patients is increasing in the past few years. Consequently, more diabetic patients are seeking orthodontic treatment than before. Diabetes mellitus is a group of metabolic diseases in which a person has high blood sugar, either because the body does not produce enough insulin or because cells do not respond to the insulin that is produced. Two fasting glucose measurements above 126 mg/dL (7.0 mmol/L) are considered diagnostic for diabetes mellitus. According to U.S. Department of Health and Human Services, 14.7 million people are diagnosed with diabetes from the age of 20 to 65. Diabetes is prevalent not only in adults but also in youth. During 2002–2005, 15,600 youth (younger than 20 years of age) were newly diagnosed with type 1 diabetes annually in the USA. Although there has been some conflicting evidence, diabetic patients seem to be more prone to infection and delayed healing after surgery. Furthermore, some animal studies report that diabetes interferes with the process of osseointegration [15–19].

Clinical application and prognosis of implants in healthy patients have been studied extensively and long-term success of prosthodontic implant has been documented. However, it is not known whether diabetes increases risk of mini-implant failure.

In diabetic patients, comparative study between surface-treated implants and machine-surfaced implants has not been performed. This study aimed to investigate effect of surface-treated orthodontic mini-implants in diabetic patients. Four weeks after diabetic induction, mini-implants were placed. The mini-implants were removed after four weeks of healing period. Osseointegration on both the surface-treated and smooth surface mini-implants was examined.

## 2. Materials and Methods

### 2.1. Subjects and Induction of Diabetes

Streptozotocin and alloxan, having specific cytotoxic effects on pancreatic beta-cells, are widely used to induce diabetes mellitus in animal studies. There have been many studies to induce diabetes in rats [20, 21]. But few studies were performed in rabbits and this study was novel in inducing diabetes in rabbits. There was trial and error when finding the dosage of alloxan and timing of glucose injection.

**Figure 1:**
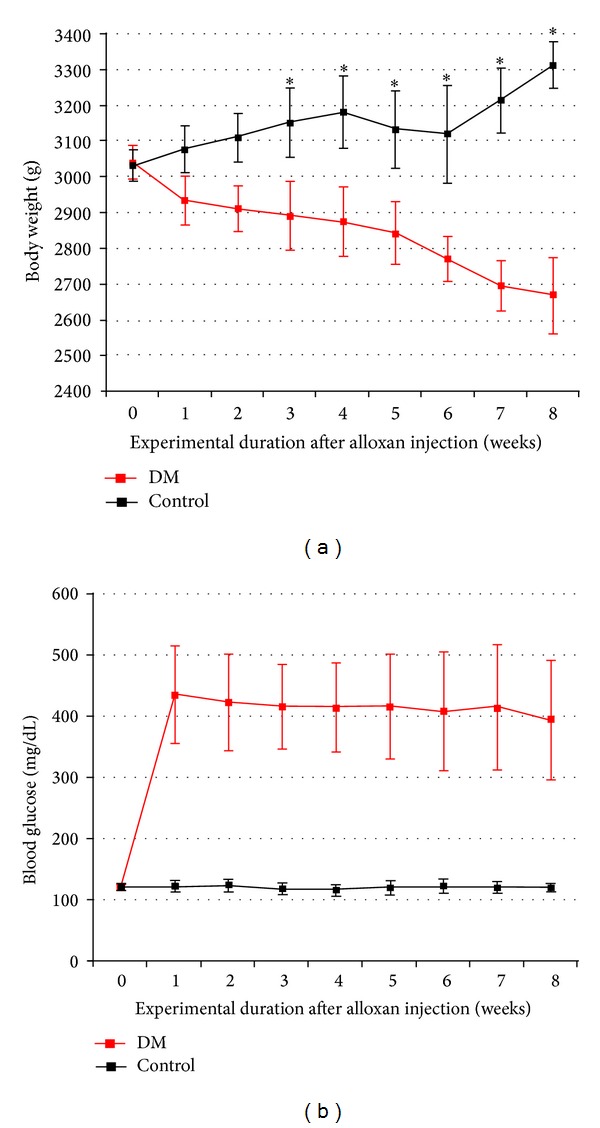
Comparison between the changes of the rabbit's weight in the control (black) and diabetic (red) groups throughout the 8-week experimental period. (a) An asterisk (∗) represents a significant difference (*P* < 0.05). (b) The line graph represents the blood glucose levels for the control (black) and diabetic (red) groups. The blood glucose level in the diabetic group increased significantly 1 week after injection of alloxan monohydrate and remained increased for the rest of the experimental period (*P* < 0.05).

After the pilot animal study to clarify the adjustment of diabetic induction, 12 New Zealand white rabbits, weighing approximately 3 kg, were used. Eighteen rabbits were assigned to diabetic group and single intravenous injection of 150 mg/kg body weight 10% alloxan monohydrate (Sigma Co., St. Louis, USA) into a marginal aural vein [20, 21]. After injection of alloxan, 20 mL of 5% glucose (JW Pharmaceutical, Seoul, Republic of Korea) was injected 5 times subcutaneously to prevent hypoglycemic shock. Additional glucose injection was given to rabbits that denied feeding for three days. Blood glucose was monitored by the glucose-oxidase method one week after the injection of alloxan. Tail-nicked blood samples were obtained and rabbits were also monitored for weight loss or gain as an indicator of overall health weekly. If the glucose level was over 200 mg/dL, a diagnosis of diabetes was made [20, 21]. Blood glucose levels in diabetic rabbits were more than 300 mg/dL throughout the entire experiment (Figure 1). Six healthy controls and six diabetic rabbits were used. Experiment protocol was approved by the Institutional Animal Care and Use Committee (The Catholic University of Korea, St. Mary's Hospital, Seoul, Republic of Korea) (CUMC-2010-0094-04). This study was performed by one examiner for reproducibility for the quantitative evaluation.

### 2.2. Surgical Procedures

A total of 48 orthodontic mini-implants were used in this study (24 SLA surface-treated, 24 smooth surface implants). The mini-implants were 1.8 mm in diameter and 8.5 mm in length. They were two-component system composed of screw and head portion (threaded portion 6.5 mm) (Cimplant Co., Seoul, Republic of Korea) [22]. The mini-implants were self-tapping and dull-pitched modified cylinder type and they were the same for both diabetic and control groups except for the surface treatment of one group.

**Figure 2:**
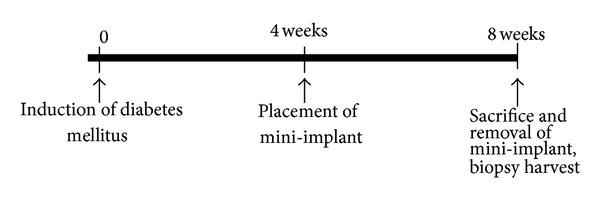
Chronologic sequence of the study.

Because blood glucose level increased significantly after injection and steadily maintained during 4 weeks and body weight decreased in diabetic group from 3 weeks (Figure 2); the implants were placed on six healthy controls and six diabetic rabbits 4 weeks after the induction of diabetes. Two anesthetics were intramuscularly administered for general anesthesia, Tiletamine-Zolazepam (10 mg/kg, Zoletil, Virbac Korea Co., Seoul, Republic of Korea) and Xylazine (2 mg/kg, Rompun, Bayer Korea, Seoul, Republic of Korea). To obtain local anesthesia and hemostasis, 1.8 mL of local anesthetic (2% lidocaine with 1 : 100,000 epinephrine) was injected in the surgical site. The surgical area was shaved and disinfected with potadine solution. The dissection was performed with a number 15 blade through skin and subcutaneous tissue to the periosteum and fascia. A periosteal elevator was used to expose the tibia.

**Figure 3:**
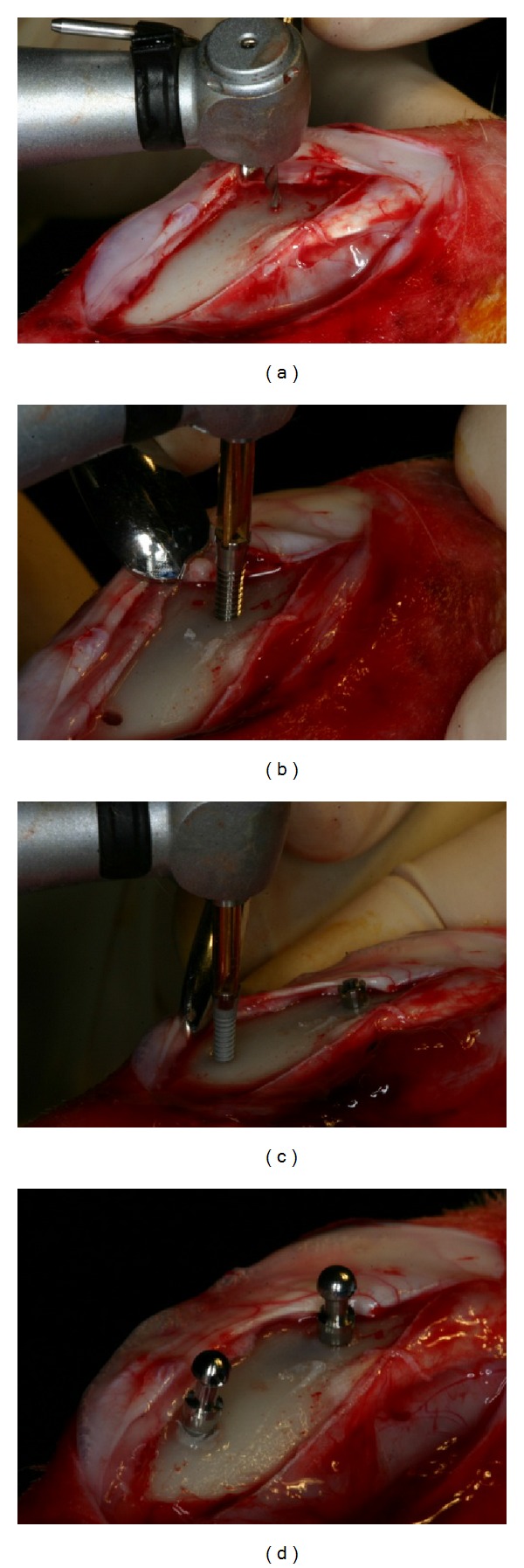
Orthodontic mini-implants placed in rabbit tibia ((a) predrilling with guide drill of 1.5 mm width. (b) The machined surface mini-implants being inserted. (c) SLA surface-treated mini-implants being inserted. (d) The head part being connected on the screw part of mini-implants.).

Mini-implants were placed according to the randomized balanced complete block design to maintain sufficient distance between each other and minimize the position difference and variation [13, 23]. Predrilling was carried out with 1.5 mm diameter guide drill under profuse irrigation to penetrate 3.5 mm into the bone. Mini-implants were placed to penetrate through the first cortical layer and reach approximately 6.5 mm [13]. A surgical engine (Elcomed SA 200 C, W&H, Burmoos, Austria) was used to record insertion torque in every 0.125 second during insertion of mini-implants. Insertion depth was controlled by fully embedding surface-treated area into bone [23, 24]. After placement, mini-implant head was connected (Figure 3). Periosteum and muscle were closed in separate layers using absorbable sutures. Analgesics (Ketoprofen 1 mg/kg, q.d.) and antibiotics (Gentamicin 4 mg/kg, q.d.) were subcutaneously administered for 3 days.

### 2.3. Removal of Mini-Implants and Histomorphometric Evaluation

Six diabetic and six control rabbits were randomly sacrificed following four weeks of healing period with overdose of anesthetics. Bone metabolism in rabbit is three times faster than human and four weeks in rabbit correspond to 3 months in human.

A total of 32 implants were removed from 4 control rabbits and 4 diabetic rabbits. Removal torque was measured with the surgical engine, during counterclockwise rotation. For mechanical analysis, torque was measured continuously during insertion and removal of mini-implants, and maximum torque was extracted from these measures. Total insertion energy was calculated during placement to maximum torque point. Total removal energy was calculated from maximum torque point to complete removal.

Specimens for the histomorphometric evaluation were prepared with 16 mini-implants around tibia in remaining two control rabbits and 2 diabetic rabbits. Tibia containing mini-implants were dehydrated in step gradients of ethanol (70%, 80%, 90%, and 100%) and infiltrated and embedded in a mixture of ethanol and Technovit 7200 resin (EXAKT GmbH, Germany). Samples were sectioned by EXAKT diamond cutting system after hardening of resin and the sections were polished to a final thickness of 40 ± 5 *μ*m by EXAKT grinding system. The specimens were then stained with hematoxylin-eosin and investigated by light microscopy. CCD camera (SPOT Insight 2 Mp scientific CCD digital Camera system, DIAGNOSTIC instrument, Inc., USA), attached to light microscope (BX51, OLYMPUS, Japan), was used to obtain images. Digitized images were evaluated histomorphometrically using SPOT Software V 4.0 (Diagnostic Instrument, USA) and Image Pro plus (Media Cybernetics, USA). The percentage of bone to implant contact (BIC %) was calculated as total BIC divided by total circumference of mini-implant × 100.

### 2.4. Statistical Analysis

The amount of total energy during placement and removal was calculated using a computer program [25]. Statistical analysis was performed with the language* R*. Two-way ANOVA (analysis of variance) was calculated to compare the results according to the presence of diabetes and surface treatment. Mann-Whitney test for nonparametric statistics was performed for the analysis of BIC. A significant *P* value was set at <0.05.

## 3. Results

### 3.1. Average Body Weight and Blood Glucose Level

Figure 1(a) shows the weights of the rabbits (mean ± standard deviation (SD)) in both groups. The diabetic group showed a significant decrease in weight from 3 weeks in comparison with the control group (*P* < 0.05). One week after injection of 10% alloxan monohydrate, the glucose-oxidase method showed that the blood glucose levels showed hyperglycemic state throughout the entire experiment and sustained weight loss was observed in diabetic rabbits as in previous studies [20, 21]. No significant inflammation was found at the surgical site.

### 3.2. Torque and Energy during Insertion and Removal

In both diabetic and control groups, all the mini-implants remained stable and did not fail until removal.

There was no significant difference between diabetic and control groups in maximum insertion torque, regardless of surface treatment. Total insertion energy was higher in control group than diabetic group. In maximum removal torque, no significant difference was found between diabetic and control groups. Total removal energy was greater in diabetic group but difference was not clinically significant. In the diabetic group, maximum removal torque and total removal energy were significantly higher in the surface-treated group than in the smooth surface group. In the healthy control group, however, there was no significant difference in total removal energy between the surface-treated group and the smooth surface group.

### 3.3. Histomorphometric Evaluation (Figure 4)

BIC was increased in the control group compared with the diabetic group without statistical significance. Similarly, in both control and diabetic groups, BIC was increased in surface-treated group compared with non-surface-treated group but there was no significantly statistical difference (Table 1).

## 4. Discussion

Previous literatures have reported that diabetic state led to more bone loss and reduced bone formation. Chronic hyperglycemia due to insulin deficiency state is known to suppress bone formation. Long-term increase in blood glucose concentration alters the response to parathyroid hormone that suppresses osteoblast differentiation and regulates the metabolism of calcium and phosphate [26]. Hyperglycaemia leads to increased formation and accumulation of advanced glycation end products (AGEs) in the blood. These molecules develop microvascular complications and reduce the number of osteoblasts and the level of osteocalcin and hence have an effect on bone matrix and slow bone formation [27–29]. The diabetic hyperglycemic state also has a negative impact on mineral deposition and bone density and delays bone healing and metabolism. Thus, diabetes increases failure rate of prosthetic implants [14–18, 30–32].

Maximum insertion and removal torque and total insertion and removal energy were used to evaluate osseointegration and stability of the mini-implant. Stress to adjacent bone during mini-implant placement was evaluated with insertion torque. Total insertion energy is the total energy recorded from the beginning of insertion to the point at which the maximum insertion torque is reached. The pressure exceeding the normal limit can cause complications such as blood circulation blockage and microfracture [33]. Previous studies have shown that total insertion energy should be in adequate range. Removal torque was used to measure mechanical interlocking between bone and implant surface. Contact area and removal torque between bone and implant were increased over time and highly correlated [34]. To minimize measuring error, a surgical engine, which can record the torque in every 0.125 second during insertion and removal of mini-implants, was used to measure maximum insertion and removal torque and total insertion and removal energy.

In maximum insertion torque, there was no significant difference between diabetic and control groups. Total insertion energy, however, was higher in the control group than the diabetic group. Total insertion energy is the area below the graph of continuous torque measured during placement. The slope of the graph to maximum insertion torque is steeper in control group than diabetic group. Therefore, total insertion energy, the area below the steep graph, is greater in control group than diabetic group. The stress applied to adjacent bone at the time of implantation is thought to be less in diabetic group due to compromised bone quality.

Total removal energy was greater in diabetic group but the amount of difference was not clinically significant. No significant difference was found in maximum removal torque between diabetic and control groups. These findings were interesting, since the authors had anticipated that maximum removal torque and total removal energy would be higher in the normal group than in the diabetic group. This result can be interpreted that there is not much difference between control and diabetic groups in mechanical interlocking between bone and implant surface.

Currently, most of available orthodontic mini-implants are not surface-treated. In this study, the difference in osseointegration between the surface-treated group and the non-surface-treated group was investigated through the placement of implants in healthy controls and diabetic rabbits, respectively.

Since the mini-implants in this study are in same shape, the amount of load to surrounding bone during placement was not significantly different; thus total insertion energy was similar between smooth surface group and SLA treated group. And because the moment of disosseointegration is maintained very shortly and decreases rapidly to zero, degree of osseointegration is represented in maximum removal torque rather than total removal energy. The maximum removal torque of the surface-treated group was significantly higher than that of the smooth surface group in both the diabetic and control groups. Surface-treated mini-implants showed more resistance by showing higher maximum removal torque in both diabetic (6.13 ± Ncm > 3.94 ± Ncm) and control groups (5.31 ± Ncm > 3.75 ± Ncm). This result means that the surface treatment enhances the osseointegration of mini-implants even in a diabetic patient. The result is based on four- week follow-up of data after placement. Bone remodeling period in the rabbit is about one- third of that in humans. Thus, 4 weeks in rabbit indicates 3 months in human.

Both maximum removal torque and total removal energy were greater in SLA mini-implants than smooth surface mini-implants in diabetic group. Total removal energy is the total energy recorded from the point at which the maximum removal torque is reached to the end of the removal procedure. This was higher in SLA treated mini-implant than smooth surface mini-implants in diabetic group. Therefore, in diabetic patient, SLA treated mini-implants can be recommended.

In a previous study where osseointegration was histometrically analyzed 12 weeks after implantation, BIC had no significant difference between diabetic and normal groups [35]. This result is in accordance with our study. However, small sample size due to high mortality rate of rabbits during diabetes induction may be one of the reasons that BIC is not being significantly different. Another reason can be wide range of standard deviation resulting in less accurate BIC measurement. Further studies need to be conducted regarding BIC measurement. Low BIC values can be considered as anatomic limitation of rabbit model. Rabbit tibia is composed of cortical bone and the rest is bone marrow. The tibia in rabbit is a site more abundant in bone marrow than in rats.

Another limitation of this study is that the mini-implants were not loaded. Since the mini-implants were not loaded during the healing process, it can be assumed that the bone healing showed no significant difference at the interface between the mini-implant and bone in the diabetic group or the control group. Further study needs to be performed when orthodontic forces are applied on mini-implants. Moreover, type 1 diabetes was induced in this study, whereas type 2 diabetes is more clinically prevalent. In prolonged type 2 diabetes, however, the insulin deficiency can be advanced as in type 1 diabetes, so the result of this study may have relevance to many clinical situations.

## 5. Conclusion

To understand the effective use of orthodontic mini-implants in the diabetic patient, a study was performed comparing normal rabbits to rabbits with intentionally induced diabetes. It can be concluded that the use of orthodontic mini-implants in a diabetic patient is likely to show results similar to the healthy patient. Various literatures reported that surface-treated mini-implants had improved osseointegration than smooth surface mini-implants. This applies to diabetic state as well in terms of surface treatment.

In conclusion, diabetes did not interfere with success of orthodontic mini-implants. Success rate of surface-treated mini-implants was higher.

## Figures and Tables

**Figure 4:**
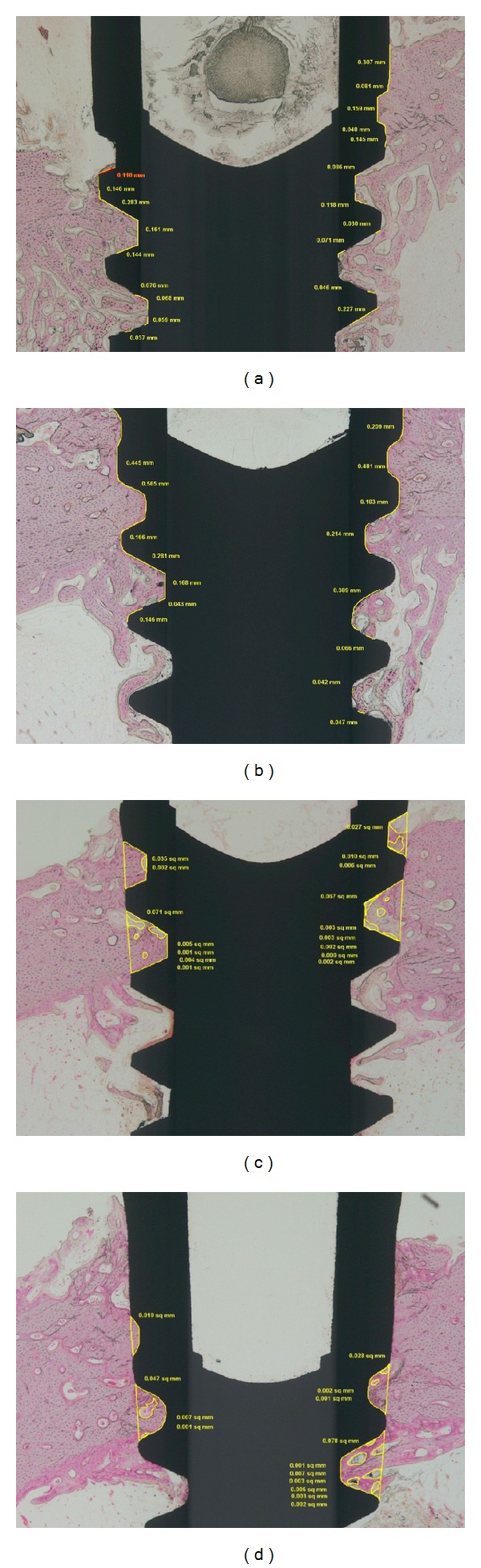
Microscopic photographs of mini-implants 4 weeks after placement (hematoxylin-eosin staining). (a) ×40; machined surface mini-implant in control group; (b) ×40; SLA treated mini-implant in control group; (c) ×40; machined surface mini-implant in DM group; (d) ×40; SLA treated mini-implant in DM group. Yellow marking means bone contact measurement.

**Table 1:** Maximum torque (Ncm), total energy (J), and BIC (%).

		DM	Type of mini-implant (mean ± SD)		Significance
			Control	SLA	
*N* = 8 per group	Maximum insertion torque (Ncm)	DM	11.63 ± 4.39	11.06 ± 5.19	Control *≓* SLA (*P* = 0.410) Normal *≓* DM (*P* = 0.460)
		Normal	13.31 ± 2.75	11.50 ± 3.26	
	Total insertion energy (J)	DM	1.64 ± 0.55	1.27 ± 0.54	Control *≓* SLA (*P* = 0.066) **Normal > DM** (**P** = 0.027)*
		Normal	1.95 ± 0.34	1.70 ± 0.35	
	Maximum removal torque (Ncm)	DM	3.94 ± 1.05	6.13 ± 2.30	**Control < SLA** (**P** = 0.001)^†^ Normal *≓* DM (*P* = 0.332)
		Normal	3.75 ± 0.85	5.31 ± 1.07	
	Total removal energy (J)	DM	0.74 ± 0.27	0.96 ± 0.61	Control *≓* SLA (*P* = 0.445) **Normal < DM** (**P** = 0.018)*
		Normal	0.56 ± 0.13	0.53 ± 0.11	

*N* = 4 per group	BIC (%)	DM	13.21 ± 5.46	14.77 ± 7.67	Control *≓* SLA (*P* = 0.798) Normal *≓* DM (*P* = 0.161)
		Normal	17.93 ± 6.71	19.48 ± 7.67	

DM: diabetes mellitus; Ncm: newton per centimeter; SLA: sandblasted with large grit and acid etched; significance: *P < 0.05; ^†^ *P* < 0.01; BIC: bone to implant contact ratio.
